# Establishing the characteristics of an effective pharmacogenetic test for clozapine-induced agranulocytosis

**DOI:** 10.1038/tpj.2015.5

**Published:** 2015-03-03

**Authors:** M Verbelen, D A Collier, D Cohen, J H MacCabe, C M Lewis

**Affiliations:** 1 SGDP Centre, Institute of Psychiatry, Psychology & Neuroscience, King's College London, London, UK; 2 Discovery Neuroscience Research, Eli Lilly and Company Ltd, Lilly Research Laboratories, Erl Wood Manor, Surrey, UK; 3 Department of Severe Mental Illness, Mental Health Care Organization North-Holland North, Heerhugowaard, The Netherlands; 4 Department of Psychosis Studies, Institute of Psychiatry, Psychology & Neuroscience, King's College London, London, UK; 5 Department of Medical and Molecular Genetics, King's College London, London, UK

## Abstract

Clozapine is the only evidence-based therapy for treatment-resistant schizophrenia, but it induces agranulocytosis, a rare but potentially fatal haematological adverse reaction, in less than 1% of users. To improve safety, the drug is subject to mandatory haematological monitoring throughout the course of treatment, which is burdensome for the patient and one of the main reasons clozapine is underused. Therefore, a pharmacogenetic test is clinically useful if it identifies a group of patients for whom the agranulocytosis risk is low enough to alleviate monitoring requirements. Assuming a genotypic marker stratifies patients into a high-risk and a low-risk group, we explore the relationship between test sensitivity, group size and agranulocytosis risk. High sensitivity minimizes the agranulocytosis risk in the low-risk group and is essential for clinical utility, in particular in combination with a small high-risk group.

## Introduction

Although there are 22 FDA-approved antipsychotics used to treat schizophrenia, around 30% of schizophrenia patients do not respond to drugs other than clozapine.^1^ Clozapine has superior efficacy for positive symptoms in these treatment-resistant patients^2^ and may improve negative symptoms.^3^ Furthermore, clozapine reduces suicidal behaviour especially when compared with first generation antipsychotics and overall mortality at population level.^4, 5, 6, 7, 8^

Despite its proven efficacy, the clinical use of clozapine is limited by the risk of agranulocytosis, a rare but potentially fatal adverse drug reaction, characterized by the acute loss of neutrophils in circulating blood. Agranulocytosis is defined as an absolute neutrophil count (ANC) of less than 500 cells mm^−3^ blood. Shortly after clozapine was introduced in Europe in the 1970s, it was withdrawn from the market when 17 cases of agranulocytosis were reported in Finland, of which 8 were fatal.^9^ In 1990, clozapine was reintroduced after its superiority over chlorpromazine for the treatment of refractory schizophrenia was shown.^10^ However, its use was restricted in most Western countries to treatment of refractory patients, that is, patients who have not improved on at least two different antipsychotics.^2, 11, 12, 13^

To prevent agranulocytosis by detecting a fall in ANC, patients treated with clozapine are subject to compulsory haematological monitoring. In Europe, the full white blood cell count and ANC are monitored weekly for the first 18 weeks of treatment and every 4 weeks thereafter for the duration of the treatment.^14^ If at any time during treatment the white blood cell count falls below 3000 cells mm^−3^ or the ANC below 1500 cells mm^−3^, clozapine should be discontinued immediately and these patients should not be treated with clozapine again except in a controlled setting.^15, 16, 17^ Although the obligatory monitoring has the benefit of regular contact with a health-care professional, it is an invasive procedure and can be a burden for the patient. Moreover, some patients decline to take clozapine because of the monitoring requirement.^18^

As agranulocytosis can develop within 2–5 days, even weekly monitoring cannot guarantee timely detection in all cases.^19^ The incidence of agranulocytosis induced by clozapine varies between 0.38 and 0.8%, with approximately 80% of cases occurring within the first 18 weeks.^20, 21, 22, 23^ The incidence of agranulocytosis decreases from 0.7% in the first year, to 0.07% or lower in the second year of treatment.^24, 25^ Few cases occur later in the course of treatment, but the risk does not fully disappear. In 2–4% of patients, agranulocytosis is fatal, which corresponds to an overall mortality rate of about 1–3 in 10 000 patients on clozapine.^26^ However, most patients recover completely from agranulocytosis with no haematological consequences.^23, 24, 27^

In spite of its therapeutic advantages with respect to its efficacy in treatment-resistant schizophrenia, clozapine is underused, mainly owing to the risk of severe adverse events, primarily agranulocytosis and the mandatory haematological monitoring.^28^ Around 30% of schizophrenia patients meet the indications for clozapine treatment, but the market share of clozapine, which is now a generic drug, was less than 5% in 2010 in the US.^2^

A pharmacogenetic test for clozapine-induced agranulocytosis could greatly improve the burden of haematological monitoring if the monitoring requirements could be made less onerous, or be time-limited, for the majority of patients with a low genetic risk for agranulocytosis. Not only would this make clozapine treatment more acceptable for the patient, it would also save considerable health-care resources. On the other hand, the patients who are at a higher risk of developing agranulocytosis could be monitored more frequently or, if the risk is very high, not exposed to clozapine at all.

Pharmacogenetic research of clozapine-induced agranulocytosis has focused on candidate genes in case–control studies. Several associations with human leukocyte antigen (HLA) alleles have been reported, as well as associations with the tumour necrosis factor and N-ribosyldihydronicotin-amide quinone oxido-reductase 2 (NQO2) genes.^29, 30^ However, few of these findings have been replicated, and the majority of these pharmacogenetic studies suffered from typical candidate gene study issues, namely small sample sizes and inadequate correction for multiple testing. The most promising finding was that the HLA-DQB1 6672G>C polymorphism was associated with clozapine-induced agranulocytosis, with an odds ratio of 16.9.^31^ A pharmacogenetic test based on this polymorphism has been marketed, but owing to low sensitivity (21.5%), it failed to be a commercial or clinical success.^29, 32^ In the first genome-wide association study, amino acid changes in HLA-DQB1 (126Q) and HLA-B (158T) were associated with clozapine-induced agranulocytosis with more modest odds ratios of 0.19 and 3.11, respectively.^33^

Here, we investigate the required properties of a clinically useful pharmacogenetic test that could stratify clozapine users with regards to their agranulocytosis risk as described above.

## Methods

We assume that the genetic test divides patients into two groups with different levels of agranulocytosis risk, and that the low-risk (LR) group contains a higher proportion of patients than the high-risk (HR) group. When comparing the outcome of a pharmacogenetic agranulocytosis test with the actual agranulocytosis status, the following scenarios can occur (Table 1):

- True positive: A patient who does develop agranulocytosis is correctly identified as HR. This scenario has probability *a*.
- False positive: A patient who does not develop agranulocytosis is wrongly identified as HR. This scenario has probability *b*.
- False negative: A patient who does develop agranulocytosis is wrongly identified as LR. This scenario has probability *c*.
- True negative: A patient who does not develop agranulocytosis is correctly identified as LR. This scenario has probability *d*.

If the incidence of clozapine-induced agranulocytosis is a known parameter *k*, the agranulocytosis risk regardless of test outcome (*a*+*c*) equals *k* and the probability of not getting agranulocytosis (*b*+*d*) is 1-*k*.

Assuming that *k* is known, two of the following parameters need to be fixed to calculate the probabilities in each cell of Table 1:

- The proportion of patients in the HR group (*x*) or the proportion of patients in the LR group (1-*x*).
- The sensitivity of the test, which is the proportion of correctly classified agranulocytosis cases. In Table 1, 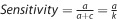
- The specificity of the test, which is the proportion of correctly classified agranulocytosis-free patients. In Table 1, 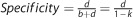

The proportion of patients in each risk group (*x,* 1-*x*) is relevant to this study, as we want to justify a more lenient monitoring schedule for the LR group. The larger the LR group, the more patients on clozapine will benefit from monitoring regime changes. Assuming a single locus test, the size of the two risk groups depends on the allele frequency of the test marker. Hence, we use the proportion of patients in the HR group (*x*) and test sensitivity (*s*) to study the cell probabilities in Table 1.

Of primary interest is the agranulocytosis risk in the LR group, as these are the patients for whom the haematological monitoring rules could be relaxed, and this outcome corresponds to the complement of the negative predictive value (NPV), being the proportion of test negative or LR patients who do not develop agranulocytosis. Therefore, we investigate the relationship between agranulocytosis risk in the LR group, test sensitivity and the size of the HR group. The agranulocytosis risk in the LR group is given by


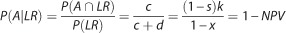


where *A* stands for developing agranulocytosis. Thus, the agranulocytosis risk in the LR group decreases as test sensitivity increases or as the HR group becomes smaller, which corresponds to the LR group getting larger.

As a secondary outcome, we study the agranulocytosis risk in the HR group, which corresponds to the positive predictive value (PPV) or the proportion of test positive patients who are true agranulocytosis cases, and is given by


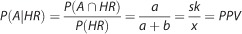


In the HR group, the agranulocytosis risk increases when sensitivity rises or the proportion of patients in the HR group decreases.

By definition, the agranulocytosis risk in the HR group must be larger than in the LR group, so


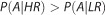


or expressed in terms of sensitivity and size of the HR group


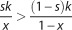


which reduces to *s>x.* In other words, sensitivity must be larger than the proportion of patients in the HR group.

We explore the relationship between different parameters, focusing on sensitivity (*s*), and the proportion of patients assigned to the HR group (*x*), in the context of a genetic test to predict the risk of clozapine-induced agranulocytosis. Furthermore, we develop guidelines for a test that divides the population into a LR and HR group, assuming the total risk of developing clozapine-induced agranulocytosis (*k*) is 0.8%.^11^ We also assess how the pharmacogenetic test based on the HLA-DQB1 6672G>C polymorphism performs under this framework.^31^

## Results

**Figure 1:**
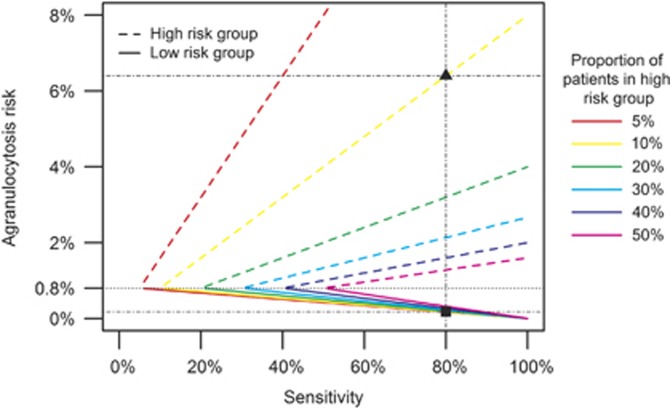
Agranulocytosis risk in LR and HR groups by sensitivity, for different HR group sizes between 5 and 50%. The ▪ and ▴ indicate the agranulocytosis risk in the LR and HR groups, respectively, for a test with 80% sensitivity and 10% of patients in the HR group.

The key parameters for a clinically effective test, that is, a test that minimizes the agranulocytosis risk in the LR group, are high sensitivity and to a lesser extent a small proportion of patients assigned to the HR group. Figure 1 shows that to obtain a low agranulocytosis risk in the LR group (solid lines), with a concomitant high risk in the HR group (dotted lines), a test should be highly sensitive. Also, for a given sensitivity, a smaller HR group corresponds to lower agranulocytosis risk in the LR group and a higher agranulocytosis risk in the HR group (Figure 1 and Table 2). The lower the sensitivity of a test is, the smaller the difference between the agranulocytosis risks in both groups. When the sensitivity is equal to the size of the HR group, the risk in the two groups are the same and equal to the overall agranulocytosis risk of 0.8%. A test with sensitivity close to the proportion of patients in the HR group would thus be irrelevant.

**Figure 2:**
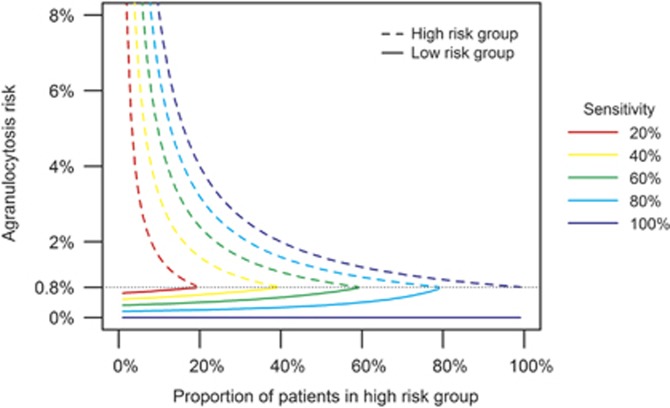
Agranulocytosis risk in LR and HR groups by proportion of patients that classified as HR, for different sensitivity values between 20 and 100%.

Exploration of the relationship between agranulocytosis risk and the proportion of patients in the HR group confirms that a smaller HR group gives rise to a lower agranulocytosis risk in the LR group (Figure 2). The risk in the HR group increases steeply when the size of that group is close to zero. For a given size of the HR group, high sensitivity leads to a low agranulocytosis risk in the LR group and a high risk in the HR group (Figure 2 and Table 3). As in Figure 1, the risk curves in Figure 2 meet at 0.8% agranulocytosis risk when the proportion of patients in the HR group is equal to the sensitivity (except for 100% sensitivity where the agranulocytosis risk in the LR group is zero as all agranulocytosis cases are detected by the test).

**Figure 3:**
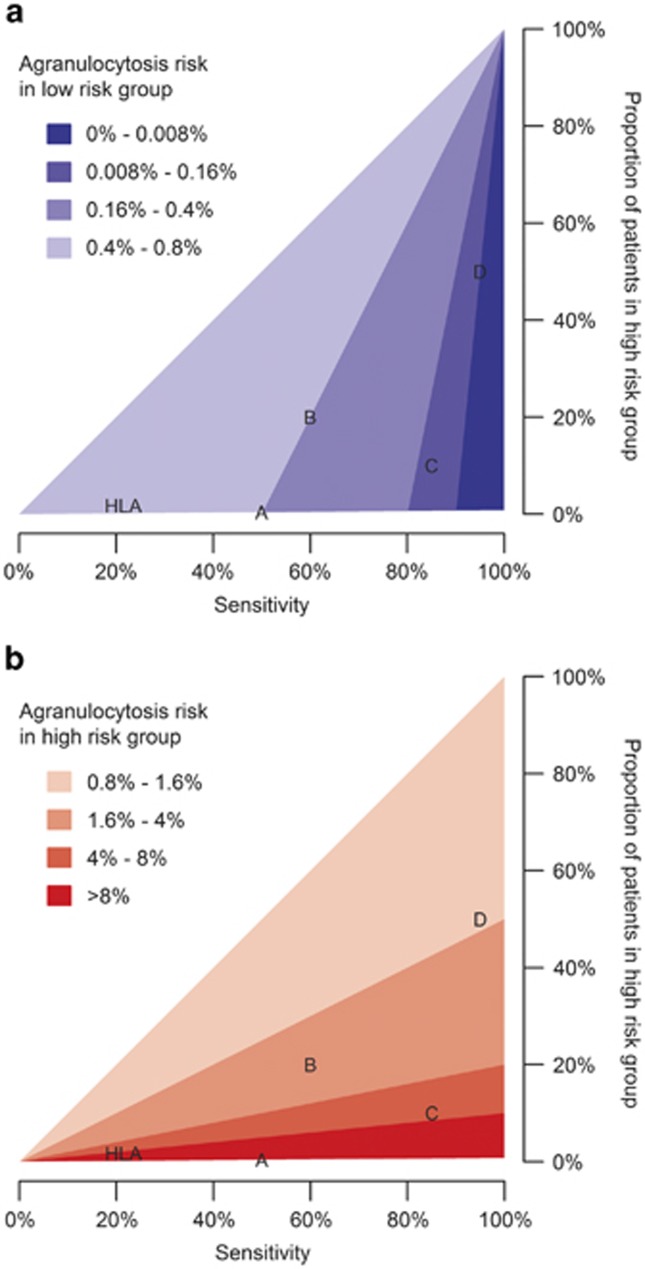
Agranulocytosis risk in (**a**) LR group and (**b**) HR group by sensitivity and proportion of patients in the HR group. In both panels, darker colours represent the desired outcomes of low risk in the LR group and high risk in the HR group. The letters indicate the position of hypothetical tests A, B, C and D; HLA indicates the position of the HLA-DQB1 6672G>C-based test.

A simultaneous assessment of sensitivity and HR group size shows that test sensitivity controls the reduction in agranulocytosis risk seen in the LR group, with high sensitivity leading to low agranulocytosis risk (Figure 3a). High sensitivity implies that most agranulocytosis cases are identified by the genetic test and classified as HR. By consequence, nearly all patients in the LR group do not develop agranulocytosis, and hence the risk in that group is low. For example, if we need the agranulocytosis risk in the LR group to be half the population risk, (that is, ⩽0.4%), the test sensitivity must be at least 50.2%. To achieve a stratification where the LR group is at one-fifth of the average agranulocytosis risk, sensitivity greater than 80.1% is required. A small HR group contributes to a low agranulocytosis risk in the LR group by preventing true LR patients from being wrongly classified as HR. A large number of true LR patients maximizes the denominator of the agranulocytosis risk in the LR group, and thus minimizes the risk itself.

The agranulocytosis risk in the HR group depends largely on the size of the HR group (Figure 3b). In a smaller HR group, the ratio of true HR patients versus patients incorrectly classified as HR is larger, and so the agranulocytosis risk in the HR group is larger. High sensitivity increases the number of true HR patients and in that way leads to a high agranulocytosis risk in this group, but even in the ideal scenario of maximum sensitivity, the proportion of true HR patients is limited to 0.8%.

High sensitivity and a small HR group lead to an effective test with the small group of HR patients requiring more frequent monitoring, whereas the majority of patients are assigned to the LR group, which has substantially reduced agranulocytosis risk and could therefore be monitored less frequently.

To comment on the clinical utility of a pharmacogenetic test, an agranulocytosis risk that is acceptable without monitoring must be determined. We propose that an agranulocytosis risk of 0.13% is acceptable, because this corresponds to the risk conferred by the antipsychotic chlorpromazine which does not have mandatory monitoring in the UK.^34, 35^ To achieve this, the sensitivity of the test must be at least 83.9%. We examine four hypothetical pharmacogenetic tests and how the outcomes affect haematological monitoring of patients (Table 4).

- Test A is only clinically relevant for the small proportion of HR patients, but owing to its low sensitivity, the agranulocytosis risk in the LR group is too high to reduce monitoring.
- Despite a higher sensitivity than test A, test B has no definite impact on the treatment of either risk group.
- The characteristics of test C result in an agranulocytosis risk in the LR group that is low enough to stop or reduce haematological monitoring for 90% of patients. This test has the largest clinical impact.
- Although the sensitivity of test D is higher than that of test C, the larger size of the HR group in test D implies that fewer patients would benefit from this test.

The pharmacogenetic agranulocytosis test using the HLA-DQB1 6672G>C polymorphism has a sensitivity of 21.5% and specificity of 98.4%.^31^ On the basis of those values and assuming an overall agranulocytosis risk of 0.8%, the test classifies 1.76% of patients as HR, with a high agranulocytosis risk of 9.66%. Conversely, the agranulocytosis risk in the LR group is 0.64%. This is a relative risk of 0.8 or a 20% reduction in risk compared with the agranulocytosis risk without genetic stratification, and exceeds our maximum acceptable agranulocytosis risk of 0.13%. Hence, the HLA-DQB1 6672G>C-based genetic test has limited use in stratifying patients in order to reduce haematological monitoring requirements for a subset of patients.

## Discussion

We have established a framework for assessing the utility of a genetic test for clozapine-induced agranulocytosis and explored the characteristics of tests that would reduce agranulocytosis risk to a level that does not require regular haematological monitoring. In particular, we show that high sensitivity is essential and that a small proportion of patients classified as HR further decreases the agranulocytosis risk in the LR group.

High sensitivity is a self-evident characteristic of a clinically useful test, but the finding that a small HR group is favourable might seem counterintuitive. One could reason that to be sure the LR group contains no agranulocytosis cases, the HR group should include all patients who are at the slightest risk of developing agranulocytosis, and that the HR group should thus be large. However, as the number of true agranulocytosis cases is very low, a large HR group would mainly contain false-positive patients. Instead of a large HR group, a useful test relies on high sensitivity to correctly classify the agranulocytosis cases as HR. A small HR group implies few false positives and many true negatives, which in turn minimizes the agranulocytosis risk in the LR group.

Instead of a single genetic locus, a pharmacogenetic test could also be based on polygenic risk scores, built from combining risk conferred by many genetic loci to identify patients at high risk. In that case, the threshold defining LR and HR groups can be varied, and the effectiveness of a test is typically measured by the area under a receiver operating characteristic curve.^36^ When the threshold is moved to increase test sensitivity, the size of the HR group will increase as well. It is not straightforward to predict the resulting change—increase or decrease—in agranulocytosis risks in the LR and HR groups, because these depend on the distribution of the polygenic risk scores. Once an appropriate polygenic score threshold has been fixed, a test can be translated easily to the framework developed here.

Pharmacogenetic tests for abacavir, carbamazepine and purine analogues in clinical use have sensitivity, specificity and NPV close to 100% (Table 5).^37, 38, 39^ However, the PPVs of these tests vary between 7.7 and 80.6%. These low PPVs are acceptable, as there are alternative treatments available for patients who test positive. In contrast, clozapine is reserved for treatment of refractory schizophrenia and no alternative drug is available. A high PPV would ensure that few patients are unnecessarily excluded from treatment, but a high NPV and consequently low agranulocytosis risk is also important to justify a reduced monitoring schedule for patients in the LR group.

No genetic test for clozapine-induced agranulocytosis currently exists. The proposed test of HLA-DQB1 6672G>C has high specificity, but low sensitivity fails to reduce the agranulocytosis risk in the LR group sufficiently that monitoring could be reduced or ceased. Candidate gene studies have failed to identify a strong, replicated genetic variant that substantially increases risk of clozapine-induced agranulocytosis.^29, 30^ The first genome-wide association study of clozapine-induced agranulocytosis detected significant associations at two HLA amino acids;^33^ at least one further study is in progress,^40^ and combined analysis of such studies may identify associated genetic variants that can be rapidly translated to clinical practice.

## Figures and Tables

**Table 1:** Classification table comparing test outcome with true agranulocytosis status

*Test result*	*Agranulocytosis*		*Total*
	*Yes*	*No*	
Positive: High risk	*a*=*sk*	*b*=*x*-*sk*	*a*+*b*=*x*
Negative: Low risk	*c*=(1-*s*) *k*	*d*=1-*x*-(1-*s*) *k*	*c*+*d*=1-*x*
Total	*a*+*c*=*k*	*b*+*d*=1-*k*	1

Cell probabilities are expressed in terms of sensitivity (*s*) and size of the HR group (*x*).

**Table 2:** Hypothetical tests with 80% sensitivity for different proportions of patients in the HR group, showing agranulocytosis risks in the LR group (1-NPV), the HR group (PPV) and specificity of the test

*Size of HR group*	*P(A|LR) (1-NPV)*	*P(A|HR) (PPV)*	*Specificity*
0.010	0.0016	0.640	0.996
0.050	0.0017	0.128	0.956
0.100	0.0018	0.064	0.906
0.200	0.0020	0.032	0.805
0.300	0.0023	0.021	0.704
0.400	0.0027	0.016	0.603
0.500	0.0032	0.013	0.502

Abbreviations: HR, high risk; LR, low risk; NPV, negative predictive value; PPV, positive predictive value.

**Table 3:** Hypothetical tests with 10% of patients in the HR group for different sensitivity values, showing agranulocytosis risks the LR group (1-NPV), in the HR group (PPV) and specificity of the test

*Sensitivity*	*P(A|LR) (1-NPV)*	*P(A|HR) (PPV)*	*Specificity*
0.200	0.0071	0.016	0.901
0.400	0.0053	0.032	0.902
0.600	0.0036	0.048	0.904
0.800	0.0018	0.064	0.906
1	0	0.080	0.907

Abbreviations: HR, high risk; LR, low risk; NPV, negative predictive value; PPV, positive predictive value.

**Table 4:** Four hypothetical pharmacogenetic tests for clozapine-induced agranulocytosis and their clinical impact

*Test*	*Sensitivity*	*Size of HR group*	*P(A|LR) (1-NPV)*	*P(A|HR) (PPV)*	*Specificity*	*Clinical impact*
A	0.500	0.004	0.004	1	1	No change in monitoring, but withhold clozapine from HR group
B	0.600	0.200	0.004	0.024	0.803	No change in monitoring
C	0.850	0.100	0.0013	0.068	0.906	Stop monitoring in LR group
D	0.950	0.500	0.0008	0.015	0.504	Stop monitoring in LR group

Abbreviations: HR, high risk; LR, low risk; NPV, negative predictive value; PPV, positive predictive value.

**Table 5:** Characteristics of pharmacogenetic tests that predict adverse drug reactions

*Drug*	*Gene*	*Test*	*Performance characteristics*	*Reference*
Abacavir	HLA-B*5701	The HLA-B*5701 allele is associated with hypersensitivity reaction. Carriers should avoid abacavir.	Sensitivity: 100% Specificity: 96.9% PPV: 47.9% NPV: 100%	^37^
Carbamazepine	HLA-B*1502	The HLA-B*1502 allele is associated with SJS-TEN in Han Chinese. Carriers should avoid carbamazepine.	Sensitivity: 98.3% Specificity: 97% PPV: 7.7% NPV: 100%	^38^
Purine analogues	TPMT	Impute 2 SNPs to identify patients with zero wildtype alleles, as they are at high risk of myelotoxicity.	Sensitivity: 100% Specificity: 99.0% PPV: 80.6% NPV: 100%	^39^

Abbreviations: HLA, human leukocyte antigen; NPV, negative predictive value; PPV, positive predictive value; SNP, single nucleotide polymorphism.
